# New Isotopes for the Treatment of Pancreatic Cancer in Collaboration With CERN: A Mini Review

**DOI:** 10.3389/fmed.2021.674656

**Published:** 2021-08-02

**Authors:** Claudia Burkhardt, Léo Bühler, David Viertl, Thierry Stora

**Affiliations:** ^1^Visceral Surgery, Surgery Department, Geneva University Hospitals, Geneva, Switzerland; ^2^Section of Medicine, Faculty of Science and Medicine, Fribourg University, Fribourg, Switzerland; ^3^Department of Nuclear Medicine and Molecular Imaging, Lausanne University Hospital, Lausanne, Switzerland; ^4^Isotope Mass Separator Online Device (ISOLDE), European Organization for Nuclear Research (CERN), Geneva, Switzerland

**Keywords:** radioisotopes, nuclear medicine, oncology, pancreatic cancer, theranostics, CERN

## Abstract

The use of radioactivity in medicine has been developed over a century. The discovery of radioisotopes and their interactions with living cells and tissue has led to the emergence of new diagnostic and therapeutic modalities. The CERN-MEDICIS infrastructure, recently inaugurated at the European Center for Nuclear Research (CERN), provides a wide range of radioisotopes of interest for diagnosis and treatment in oncology. Our objective is to draw attention to the progress made in nuclear medicine in collaboration with CERN and potential future applications, in particular for the treatment of aggressive tumors such as pancreatic adenocarcinoma, through an extensive review of literature. Fifty seven out of two hundred and ten articles, published between 1997 and 2020, were selected based on relevancy. Meetings were held with a multi-disciplinary team, including specialists in physics, biological engineering, chemistry, oncology and surgery, all actively involved in the CERN-MEDICIS project. In summary, new diagnostic, and therapeutic modalities are emerging for the treatment of pancreatic adenocarcinoma. Targeted radiotherapy or brachytherapy could be combined with existing therapies to improve the quality of life and survival of these patients. Many studies are still in the pre-clinical stage but open new paths for patients with poor prognosis.

## Introduction

Pancreatic cancer represents a significant cause of morbidity and mortality, being the 10th leading cause of death worldwide. The overall 5-year survival rate is below 5% for patients with confirmed ductal adenocarcinoma (1). Surgery remains the only curative treatment known.

The treatment strategies for locally advanced tumors may depend on whether the disease is resectable, borderline resectable, or unresectable (2).

For localized resectable pancreatic adenocarcinoma, current recommendations include surgical resection followed by 3–6 months adjuvant therapy, for example Fluoropyrimidine or Gemcitabine with radiotherapy.

Based on the M.D. Anderson criteria, borderline resectability can be defined as a tumor contact with <180 degrees circumference of the superior mesenteric artery, short-segment involvement of the common hepatic artery or short-segment occlusion of the superior mesenteric vein or portal vein (3). In cases of borderline resectability, surgery is recommended, followed by adjuvant therapy. In locally advanced unresectable disease, where the tumor is in contact with the superior mesenteric artery on more than 180 degrees circumference or when there is another vessel involvement without a feasible surgical reconstruction, neoadjuvant therapy is recommended (4).

In metastatic disease, current options include chemotherapy with Folfirinox or Gemcitabine plus Abraxane (5).

At diagnosis, 80% of patients present an unresectable disease. Amongst the patients undergoing surgical resection, 80% will develop local recurrence and/or distant metastases and die within 5 years (6). Therefore, it is necessary to identify new treatment modalities, in particular in locally advanced and/or metastatic disease, such as targeted radiotherapy or brachytherapy.

This article review aims to bring attention to the progresses in the field of nuclear medicine, in collaboration with the European Organization for Nuclear Research (CERN) and the potential future applications, in particular for the treatment of aggressive tumors such as pancreatic adenocarcinoma. Fifty seven out of two hundred and ten articles, published between 1997 and 2020, were selected based on relevancy. The following kewords were the most searched in Pubmed: brachytherapy, CERN-MEDICIS, ion beam therapy, neoadjuvant, neurotensin receptor, pancreatic cancer, radioimmunotherapy, radioisotopes, and somatostatin receptor.

## The Cern-Medicis Facility

The CERN was founded in 1954 and has 22 member states. Its main mission is to probe the fundamental structure of the universe by studying the constituents of matter, the fundamental particles, and their interactions. This research activity allowed the development of medical applications such as the positron emission tomography (PET) scanner in the early 1990s.

The Isotope mass Separator Online Device (ISOLDE) facility at CERN is dedicated to the production of radioactive ion beams for different experiments in the field of nuclear, solid-state physics and life sciences. The first experiments at ISOLDE started 50 years ago. Now over 1,300 radioactive isotopes of 70 different elements (*Z* = 2–88) with half-lives from days to milliseconds are produced at intensities up to 10^11^ atoms per microA of proton beam, using the Proton-Synchrotron Booster (PSB) at CERN (7).

ISOLDE receives about 50% of all PSB protons, from which 85% traverse the ISOLDE target without any interaction (7).

The CERN-MEDICIS (Medical Isotopes Collected from ISOLDE) facility is a project which started in 2013, aiming to recover the lost proton beam to produce radioisotopes for biomedical purposes (8).

**Figure 1:**
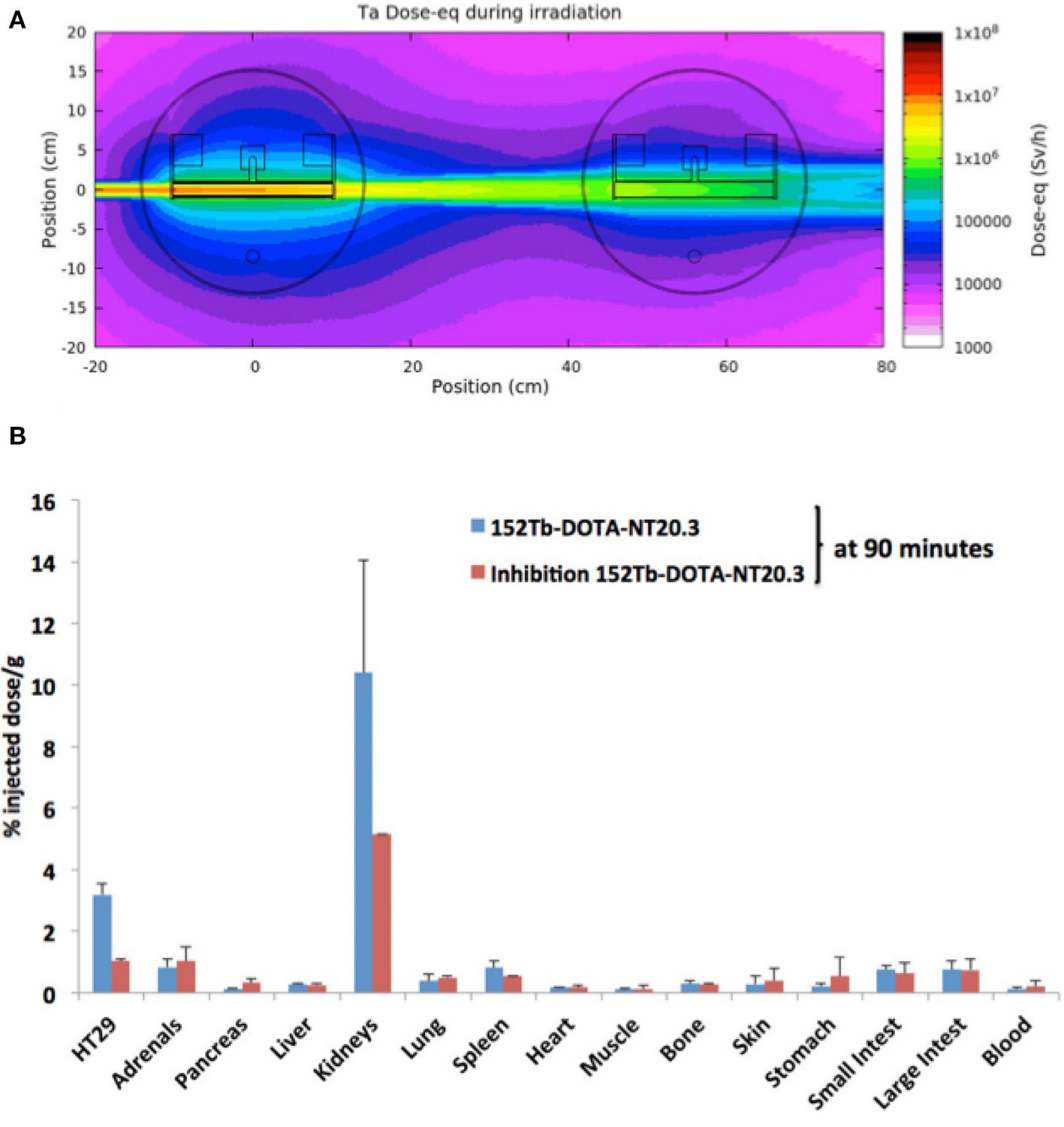
**(A)** Monte-Carlo simulations of the ISOLDE (Left) and MEDICIS (Right) targets irradiated with 1.4GeV proton beam at CERN. **(B)** Biodistribution of Tb152 bound to the neurotensin NT20.3 at 90 min post injection in mice bearing the human prostate cancer HT29 cell lines. Tumor uptake was specific as shown by the competitive inhibition experiment realized by co injection of cold radio ligand in excess. Aside the tumor the kidney also showed a strong uptake (*n* = 3).

A target consists in a small cylinder which contains different materials according to the chosen isotope production, for example ceramics or titanium foils. The CERN-MEDICIS target is placed behind the ISOLDE target, taking advantage of the remaining proton beam, which produces a variety of new elements before reaching the beam dump. Figure 1A shows a Monte Carlo simulation of the ISOLDE and MEDICIS targets.

The target is then transported through rails and handled by a robotic arm, monitored by remote computers through cameras. The target is transported to armored bunkers for isotope extraction. To produce a specific radioisotope, the elements need to undergo a physical purification by mass spectrometry, then a chemical purification by using a chelator.

Subsequently, the batches are ready to be shipped to different institutes and hospitals (9).

## General Applications

A wide range of radioisotopes can be produced by CERN-Medicis, including positron, alpha, Auger, and conversion electron emitters (10). Various chemical species, such as lanthanides, halogens, transition metals, and alkaline earth metals are available.

Table 1 shows an example of isotope production at CERN-MEDICIS and potential clinical applications.

**Table 1:** Isotope production at CERN-MEDICIS (non-exhaustive list) (7) and potential applications found in the literature (non-exhaustive list).

**Isotope**	**Half-life**	**Medical application**	**Extracted activity (Bq)**	**Example of applications [citation]**
^212^Bi	60.6 m	Alpha, beta therapy, SPECT	2.5 E9	Osteoblastic osteosarcoma (P) (7)
^213^Bi	45.6 m	Alpha, beta therapy	4.2 E8	Metastatic breast cancer therapy (P) (8)
^177^Lu	6.7 d	Beta- therapy	1.7 E8	Metastatic pancreatic adeno-carcinoma therapy (C) (9) and prostate cancer therapy (C) (10)
^166^Yb	56.7 h	Auger therapy	1.1 E10	–
^166^Ho	25.8 h	Beta therapy	6.0 E6	Radioembolisation of neuro-endocrine tumors (C) (11)
^149^Tb	4.1 h	Alpha therapy	2.4 E10	Folate receptor targeted therapy (P) (12)
^152^Tb	17.5 h	PET	2.2 E10	Metastatic neuroendocrine tumor imaging (C) (13)
^155^Tb	5.33 d	SPECT	6.8 E8	Imaging of various tumor xenografts (P) (14)
^156^Tb	5.35 d	PET	1.3 E7	–
^161^Tb	6.9 d	Beta-/Auger therapy	5.4 E6	Ovarian cancer therapy (P) (15)
^153^Sm	46.8 h	Beta therapy	1.0 E9	Skeletal metastases pain palliation (C) (16)
^140^Nd	3.4 d	PET/Auger therapy	4.0 E9	Neuroendocrine tumor imaging (P) (17)
^82^Sr	25.5 d	PET	4.0 E8	–
^89^Sr	50.5 d	Beta therapy	5.4 E8	Skeletal metastases pain palliation (C) (18)
^71^As	65.3 h	PET	1.6 E9	–
^72^As	26.0 d	PET	3.0 E9	–
^74^As	17.8 d	PET	9.0 E7	Vascular imaging of solid tumors (P) (19)
^77^As	38.8 h	Beta therapy	1.4 E9	Radioimmunotherapy targeting vascular entothelial cells in solid tumors (P) (19)
^61^Cu	3.3 h	PET	4.0 E9	Fibrosarcoma imaging (P) (20)
^64^Cu	12.7 h	PET	3.6 E9	Imaging of HER2+ breast cancer (C) (21)
^44^Sc	4 h	PET	3.2 E10	Metastatic prostate cancer imaging (C) (22)
^47^Sc	3.4 d	Beta therapy	1.2 E10	Folate receptor targeted therapy (P) (23)
^11^C	20.3 m	PET	4.2 E9	Image-guided nodal biopsy in recurrent prostate cancer (C) (24)

*(P), preclinical studies; (C), clinical studies*.

Lutetium and terbium are two particular lanthanides of interest, available at CERN-MEDICIS.

Lutetium-177 (^177^Lu) is a low-energy beta- emitting lanthanide with a long half-life of 6.65 days. The mean penetration range of the emitted beta- particles in soft tissue is short, allowing high energy delivery irradiation to small volumes, such as micrometastases or residual tumor tissue (11). Examples of *in vivo* uses include therapy with ^177^Lu-labeled PSMA (Prostate-specific membrane antigen) for the treatment of metastatic castration-resistant prostate cancer (12) as well as labeling of somatostatin analogs for the treatment of neuroendocrine tumors (13).

Terbium can form a stable compound associated with macrocyclic chelators such as DOTA (14), and has various isotopes such as ^149^Tb, ^152^Tb, ^155^Tb, and ^161^Tb.

^149^Tb is suitable for targeted alpha therapy. It decays with a half-life of 4.1 h, emitting short-range alpha-particles, gamma-rays and positrons, thus being suitable for SPECT and PET (15). ^149^Tb produced at the ISOLDE facility, has been used *in vitro* and *in vivo* in folate receptor targeted alpha-therapy studies (15). The folate receptor (FR) is expressed in ovarian and lung cancer. Mice bearing tumor with FR-positive cancer cells were injected with ^149^Tb labeled DOTA-folate conjugate (^149^Tb-cm09). Results showed significant tumor growth delay and increased survival time compared to untreated control mice. The mice showed no signs of acute kidney or liver toxicity.

^152^Tb and ^155^Tb are suitable for imaging purposes via PET and SPECT, respectively. ^152^Tb decays with a half-life of 17.5 h, through electron capture, by emitting positrons and gamma-rays.

Figure 1B represents the biodistribution of ^152^Tb bound to neurotensin after injection in mice bearing human prostate cancer cell lines at Lausanne University Hospital (CHUV).

A recent study (16) used ^152^Tb for the first time in a human, who had a metastatic neuroendocrine neoplasm. The half life of ^152^Tb allowed transportation from the ISOLDE facility over hundreds of kilometers across Europe. Results showed successful PET/CT imaging using the somatostatin analog ^152^Tb-DOTATOC, allowing the visualization of small metastases.

^155^Tb decays with a half-life of 5.33 days, through electron capture, while emitting gamma-radiation. Imaging studies have been performed in nude mice bearing tumor xenografts using a SPECT/CT scanner after injection of ^155^Tb-DOTATATE (17), showing excellent visualization of the tumor xenografts. The relatively long half-life of ^155^Tb allowed SPECT imaging several days after administration.

^161^Tb decays with a half-life of 6.9 days, emitting beta- particles and Auger electrons, and is suitable for pretreatment imaging and dosimetry through PET or SPECT (18). ^161^Tb has low photon emission, minimizing normal tissue irradiation (19), and delivers high doses to small volumes (20), which is ideal for the treatment of micrometastases or minimal residual cancer tissue. A study with ^161^Tb-labeled antibodies targeting the L1 cell adhesion molecule (L1CAM) in mice bearing ovarian cancer, showed high tumor uptake with low level of uptake in other organs. Moreover, this study showed that anti-L1CAM radioimmunotherapy is more effective with ^161^Tb than with ^177^Lu (19).

Therefore, Terbium has a high theranostic potential through its variety of radioisotopes available at the CERN-MEDICIS facility. This feature could be exploited for the therapy of aggressive cancers with limited treatment modalities, such as pancreatic adenocarcinoma.

## Applications in Pancreatic Cancer Therapy

### Targeted Radiotherapy for Pancreatic Cancer Treatment

#### Targeting Neurotensin Receptors in Pancreatic Adenocarcinoma

In pancreatic adenocarcinoma cells, there is an over-expression of neurotensin receptors, which can be targeted with radiolabelled neurotensin analogs (21). *In vitro* studies showed a high affinity of the ^68^Ga-labeled neurotensin analog (^68^Ga-DOTA-NT-20.3) for the human pancreatic ductal adenocarcinoma cell line AsPC-1 (22).

A study using a neurotensin receptor antagonist coupled to ^177^Lu (^177^Lu-3BP-227) in 6 patients with metastatic pancreatic adenocarcinoma, showed feasibility, improvement of symptoms and quality of life in all of the patients and partial response in one of the patients (23).

These studies indicate that radiolabelled neurotensin analogs are a potential new therapeutic option for the treatment of unresectable pancreatic adenocarcinoma, which could benefit from the vast isotope production at CERN-MEDICIS.

#### Targeting Somatostatin Receptors in Neuroendocrine Pancreatic Tumors

The somatostatin receptor is expressed on the cell surface of the majority of neuroendocrine tumors and can be used for imaging and targeted treatment (24).

DOTA-coupled peptides bound to the positron emitter ^68^Ga have been developed for somatostatin receptor imaging, such as DOTATOC, DOTATATE and DOTANOC, and have higher receptor affinity than Octreotide (25).

These peptides have been tested for radionuclide therapy on pancreatic neuroendocrine tumors (pNET). A retrospective trial with ^177^Lu-DOTATATE on metastatic grade 1 and 2 pNET showed to be effective with a median progression free survival of 24 months and an overall survival of 53 months (26).

Somatostatin-receptor-targeted therapy could also be used for neoadjuvant therapy to render initially inoperable pNET resectable, using ^177^Lu-DOTATATE (27) and ^90^Y-DOTATATE (28). ^90^Y is a beta-emitting radionuclide with a radiation path length of 5 mm, suitable for bulky tumors such as pancreatic tumors.

Somatostatin-receptor-targeted therapy could also be effective on pancreatic adenocarcinoma as there are somatostatin receptor subtypes which are highly expressed in exocrine pancreas adenocarcinoma (29). A preclinical study used DOTATOC coupled to the alpha-emitter Bismuth-213 (^213^Bi) on human pancreatic adenocarcinoma cells. In comparison with the beta- emitter ^177^Lu-DOTATOC, ^213^Bi-DOTATOC showed higher relative biological effectiveness and consecutively was more effective in decreasing pancreatic adenocarcinoma cell survival (30).

#### Radioimmunotherapy

The anti-mucin monoclonal antibody PAM4, is highly specific for pancreatic carcinoma. The antigen to PAM4 is MUC5AC, a secretory mucin expressed in over 85% of pancreatic carcinomas in their early stages and throughout disease progression, provinding a promising therapeutic target (31, 32).

TF10 is a humanized, PAM4-based, recombinant bispecific monoclonal antibody, which can be radiolabelled and used for pre-targeted radioimmunotherapy (33).

The tumor is first pre-targeted with an antibody construct, such as TF10, which has affinity for the tumor-associated antigen and for a radiolabelled hapten which is administered in a later phase (34). This step-by-step strategy has shown to reduce toxicity. In a pre-targeted radioimmunotherapy study with TF10-^90^Y-IMP-288, nude mice bearing human pancreatic cancer xenografts were given TF10 and then received a ^90^Y peptide later, which was as effective as radioimmunotherapy with ^90^Y-PAM4 but with less toxicity (31). Combinations with gemcitabine and dose fractionation of the pre-targeted radioimmothrapy enhanced therapeutic responses (35).

Humanized PAM4 (Clivatuzumab) labeled with ^90^Y has been proven to be active in advanced pancreatic cancer in phase I studies (36), combined with low doses of Gemcitabine which is a known radiosensitiser (37).

A phase Ib study of administering fractionated radioimmunotherapy with ^90^Y-Clivatuzumab in patients with metastatic pancreatic cancer after a median of three prior therapies, appeared to be feasible and safe, with or without Gemcitabine (38). However, the phase III trial didn't show an improvement of overall survival (39).

The fibroblast activation protein (FAP) is overexpressed in cancer-associated fibroblasts, which promotes tumor growth and progression in epithelial carcinomas such as pancreatic cancer (40). FAP-specific enzyme inhibitors (FAPI) can be labeled with ^68^Ga for imaging or with therapeutic isotopes such as ^177^Lu (41). A recent study used ^64^Cu and ^225^Ac-labeled FAPI in pancreatic cancer xenograft mouse models, revealing rapid clearance through the kidneys, high accumulation in the tumors and significant tumor growth suppression in the mice injected with ^225^Ac-FAPI (42).

Integrins are transmembrane glycoproteins that can contribute to cancer progression and be targeted for radioimmunotherapy (43). The integrin α_6_β_4_ is involved in tumor invasion and is overexpressed in pancreatic adenocarcinoma. A study using ^90^Y-labeled α_6_β_4_ integrin antibody in mice showed reduction in tumor volumes and decreased cell proliferation compared with the control group (44).

Therefore, different targets are available for endoradiotherapy in pancreatic cancer. Further studies are needed to research the effects on overall survival.

### Brachytherapy for Pancreatic Cancer Treatment

The radioactive isotopes produced by CERN-MEDICIS could also be directly implanted within the tumor tissue, using brachytherapy. After radioactive seed placement, the target tissue is continuously exposed to radiation, which produces localized tissue injury and high tumor ablation. The tumor volumes and number of implants required must be evaluated before implantation to optimize the treatment.

#### CT-Guided Percutaneous Implantation

A study on CT-guided percutaneous implantation of ^125^Iodine seeds directly in pancreatic lesions was performed in patients with stage III and IV pancreatic carcinoma, without significant adverse effects and less toxicity than standard radiotherapy (45).

Another study with ^125^I-seeds brachytherapy in patients with unresectable pancreatic cancer, showed after 2 months 70% pain relief in patients, an overall response rate (including complete and partial remission) of 65.4% and a local control rate of 88.5% (46).

A meta-analysis of 23 studies (47) concluded that ^125^I-seeds brachytherapy leads to an overall survival of 9 months in patients with advanced pancreatic cancer. When combined with other therapies such as chemotherapy, the overall survival in these patients reaches a duration of 12 months. Brachytherapy with ^125^I-seeds implantation in combination with cryoablation was found to be associated with the longest survival: up to 14 months.

Cryotherapy is performed by inserting a cryoprobe through peritoneal or retroperitoneal approach. It can also be performed on liver metastases using additional cryoprobes which are inserted through the right intercostal space. The cycles of freezing are performed once all the probes are inserted. ^125^I seed implantation is often performed following cryoablation. Studies comparing cryosurgery in combination with ^125^I seed implantation and cryosurgery alone showed higher survival rates and longer median survival in the patients undergoing combination treatment (48, 49).

#### Brachytherapy Through Endoscopy

Endoscopic ultrasonography (EUS)-guided brachytherapy has shown to be a feasible and safe treatment of unresectable pancreatic adenocarcinoma using radioactive seeds with isotopes such as Iridium-192 (^192^Ir), Palladium-103 (^103^Pd), or the most frequently used Iodine-125 (^125^I) (50). EUS-guided brachytherapy has the advantages of accurate positioning, mild injury and a shorter puncture distance than CT-guided percutaneous implantation.

In a recent retrospective clinical study, patients with stage III and IV pancreatic head adenocarcinoma underwent endoscopic brachytherapy through implantation of Iodine-125 seeds (51). Results showed no serious complications, a partial remission rate of 80% of the patients with stage III disease and an improved quality of life through an improved median Karnofsky performance status score.

Another study evaluated the results of EUS-guided brachytherapy combined with intratumoral implants for sustained delivery of 5-Fluorouracil in patients with advanced pancreatic cancer (52). A mean of 18 Iodine-125 seeds and 36 implants delivering 5-fluorouracil were inserted into the tumors. No local complications or haematologic toxicity occurred. There was a partial response in 1 out of 8 patients, a minimal response in 2 out of 8 patients and a stable disease in 3 out of 8 patients. 50% of the patients presented pain reduction and improved Karnofsky performance status score.

#### Brachytherapy Through Minimally Invasive Surgery

Encapsulated radioactive sources, such as Iodine-125 seeds, can also be placed within the tumor through minimally invasive surgery. The Da Vinci Surgical System could enable the surgeon to insert the seeds with great precision, at a safe distance to prevent unwanted irradiation, with minimal damage or complications for the patient. Few studies have been carried out yet to examine the potential benefits of robotic-assisted brachytherapy. Some studies described brachytherapy through surgery with the Da Vinci System in pigs after thoracoscopic wedge resection (53) and in patients with prostate (54) or bladder (55) cancer. There hasn't been any study using brachytherapy with the Da Vinci System in patients with pancreatic adenocarcinoma so far.

A study was performed in eight patients with unresectable pancreatic head tumors, suffering from pain of high intensity who were candidates for palliative surgery due to jaundice and/or recurrent ileus (56). They underwent perioperative high dose rate brachytherapy with ^192^Iridium implants. During the surgery, after palliative choledochoenteroanastomosis and gastrointestinal bypass using a Roux-en-Y loop, a catheter was implanted through the abdominal wall and the transverse mesocolon, to prepare the patients for later brachytherapy. Brachytherapy was initiated at the 6th post-operative day in fractionated doses of 5 Gy, by inserting temporary ^192^Ir-implants.

The patients who underwent perioperative palliative brachytherapy described more pain relief. Mean survival time was 6.7 months in the brachytherapy group, vs. 4.4 months in the group where no brachytherapy was performed (56).

A study examined the combination of palliative surgery through biliary and gastric bypass associated with surgical brachytherapy in patients with unresectable pancreatic head adenocarcinoma (57). In the group undergoing brachytherapy, during exploratory laparotomy after Kocher manoeuver, needles were implanted into the tumor and spaced at parallel intervals of 10 mm, extending at ≥5 mm beyond the margins of the mass. The needles allowed to verify positioning and were retracted if bile, blood, or pancreatic juice issued from the needle. ^125^I seeds were then injected at the location of the needles. A median of 27 seeds per patients were implanted.

No mortality occurred in the perioperative period in both groups, with or without brachytherapy and there were no significant differences in morbidity and length of hospital stay. In the group undergoing brachytherapy, partial response rate was 56 vs. 0% (*P* < 0.001) and progression was of 24 vs. 85% (*P* = 0.013). The median survival time was longer as well, corresponding to 11 months in the brachytherapy group vs. 7 months. In addition, the patients undergoing brachytherapy described an improved quality of life.

CT-guided, endoscopic, or surgical brachytherapy is therefore a valuable option for palliation of symptoms and could be combined with chemotherapy or external beam radiotherapy to improve length of survival and local tumor control.

## Conclusion

This review of literature highlights the progresses in the field of nuclear medicine for the treatment of unresectable pancreatic adenocarcinoma. As new targets for endoradiotherapy and new techniques for brachytherapy emerge, a collaboration with research facilities such as the CERN-MEDICIS infracture is needed, which provide a variety of radioisotopes. Terbium and Lutetium are two lanthanides of particular interest, with a high theranostic potential.

These new techniques could be combined to current therapies, such as chemotherapy and external beam therapy, to improve results. Further large-scale studies are necessary and multidisciplinary collaboration is essential for this purpose.

## Author Contributions

CB gathered the information, selected the articles for the article review, and wrote the article text. LB initiated and supervised the project in all its aspects and provided his knowledge and references in visceral surgery. DV allowed a visit of the Nuclear Medicine and Molecular Imaging department at CHUV and provided his knowledge and references in the field of nuclear medicine. TS allowed a visit of the CERN-MEDICIS facility and brought his knowledge and references in the field of nuclear physics. LB, DV, and TS revised the article. All authors contributed to the article and approved the submitted version.

## Conflict of Interest

The authors declare that the research was conducted in the absence of any commercial or financial relationships that could be construed as a potential conflict of interest.

## Publisher's Note

All claims expressed in this article are solely those of the authors and do not necessarily represent those of their affiliated organizations, or those of the publisher, the editors and the reviewers. Any product that may be evaluated in this article, or claim that may be made by its manufacturer, is not guaranteed or endorsed by the publisher.
